# Cochlear Implantation in Patients with Mitochondrial Gene Mutation: Decline in Speech Perception in Retrospective Long-Term Follow-Up Study

**DOI:** 10.3390/life12040482

**Published:** 2022-03-26

**Authors:** Kai Kanemoto, Akinori Kashio, Erika Ogata, Yusuke Akamatsu, Hajime Koyama, Tsukasa Uranaka, Yujiro Hoshi, Shinichi Iwasaki, Tatsuya Yamasoba

**Affiliations:** 1 Department of Otolaryngology and Head and Neck Surgery, Graduate School of Medicine, The University of Tokyo, Tokyo 113-8654, Japan; kk26921@5931.saitama-med.ac.jp (K.K.); ogataerika@g.ecc.u-tokyo.ac.jp (E.O.); aka-tky@umin.ac.jp (Y.A.); koyamah-oto@h.u-tokyo.ac.jp (H.K.); uranakat-oto@h.u-tokyo.ac.jp (T.U.); hoshi-tky@umin.ac.jp (Y.H.); tyamasoba@umin.ac.jp (T.Y.); 2 Department of Head and Neck Surgery, Saitama Medical University International Medical Center, Saitama 350-1298, Japan; 3 Department of Otolaryngology, Mitsui Memorial Hospital, Tokyo 101-8643, Japan; 4 Department of Otolaryngology and Head and Neck Surgery, Graduate School of Medicine, Nagoya City University, Nagoya 467-8601, Japan; iwashin@med.nagoya-cu.ac.jp

**Keywords:** cochlear implantation, retrocochlear dysfunction, mitochondrial gene mutations

## Abstract

Clinical evidence of the effectiveness of cochlear implantation for hearing loss with mitochondrial DNA mutation is limited. Most reports have only described short-term postoperative speech perception, which may not reflect the limitations of cochlear implantation caused by progressive retrocochlear dysfunction. The present study aimed to investigate long-term speech perception after cochlear implantation in patients with severe to profound hearing loss associated with mitochondrial DNA mutation. A retrospective chart review was performed on patients with mitochondrial DNA mutation who had undergone cochlear implantation at the Department of Otolaryngology and Head and Neck Surgery at the University of Tokyo Hospital. We extracted data on causative mutations, clinical types, clinical course, perioperative complications, and short-term and long-term postoperative speech perception. Nine patients with mitochondrial DNA mutation underwent cochlear implantation. The mean observation period was 5.5 ± 4.2 years (range, 1–13 years), and seven patients were followed for more than 3 years. Two of the seven patients who initially showed good speech perception exhibited deterioration during long-term follow-up. The absence of an acute progression of cognitive decline in patients, showing a gradual decrease in speech perception, suggests that the deterioration of speech perception was caused by progressive retrocochlear degeneration. Although most patients with mitochondrial DNA mutation maintained good speech perception for more than 3 years after cochlear implantation, retrocochlear degeneration could cause the deterioration of speech perception during long-term follow-up.

## 1. Introduction

Mitochondria play an important role in intracellular adenosine triphosphate production by oxidative phosphorylation, an essential energy source in nucleated cells. Mutations in mitochondrial DNA (mtDNA) cause dysfunction, especially in tissues with high metabolic demands. In patients with mtDNA mutations, organs that rely on aerobic energy production, such as the visual pathway, heart, central nervous system, and skeletal muscle, are primarily affected. The auditory pathway, including the cochlea, also has large energy demand; therefore, the auditory pathway is an organ that can be profoundly affected by mitochondrial disorders [1,2,3].

More than half of the patients with mtDNA mutations are affected by a hearing impairment at some time during the disease course [4,5,6]. Pathological mutations of the mtDNA have been commonly found at the transfer RNAs (tRNAs). To date, more than 90 point mutations in 21 of the 22 mitochondrial tRNA genes have been reported [7,8]. Most of these mutations result in a decreased rate of mitochondrial protein synthesis, causing a deficiency in the energy metabolism of the cell [9]. Approximately 50 mutations of the tRNA genes have been associated with deafness [10]. Associated features of hearing loss include encephalomyopathy, lactic acidosis and stroke-like episodes (MELAS syndrome), diabetes (maternally inherited diabetes and deafness, or MIDD, syndrome), ophthalmoplegia (chronic progressive external ophthalmoplegia, or CPEO, syndrome), cardiac conduction abnormalities with retinopathy and ophthalmoplegia (Kearns–Sayre syndrome), myoclonus epilepsy (myoclonus epilepsy associated with ragged-red fiber, or MERRF, syndrome), ptosis, ophthalmoplegia, gastrointestinal dysmotility, cachexia, peripheral neuropathy, and leukoencephalopathy (mitochondrial neurogastrointestinal encephalopathy, or MNGIE, syndrome). Hearing loss is usually gradual at onset, initially occurs at high frequencies, is predominantly bilaterally symmetrical, and progresses to profound. Hearing loss in patients with mitochondrial diseases is mainly attributed to cochlear dysfunction [11,12,13,14,15], but mitochondrial disorders can also affect the central nervous system, including the central auditory pathway, and can cause psychomotor regression [4,16,17,18,19]. Roesch et al. [20] conducted a systematic review of knowledge on hearing loss in genetically proven mitochondrial disease in children. A total of 75 patients from 23 studies were included in the analysis. Retrocochlear hearing loss was found more often (33 out of 75 patients) than expected. Affected genes included OPA1 in 14 patients, FDXR in seven patients, and MT-TL1 in six patients. The clinical courses of these patients, including the age of onset and disease severity, showed diverse characteristics. Takahashi et al. [21] reported histopathological examinations of human temporal bones in MELAS patients and found severe degeneration of the stria vascularis and the spiral ganglion cells. There was severe atrophy of the stria vascularis in all turns of the cochlea, and the remaining stria cells showed vacuole formation and the presence of small, dark-staining, round, and ovoid cells. Both the outer and inner hair cells were generally present, with scattered losses in the lower basal turns. In addition to these findings, the total number of spiral ganglion cells was reduced when compared with the mean values of normal newborn and age-matched control samples, representing a mild neuronal loss. Many spiral ganglion cells showed varying degrees of degenerative change, as evidenced by faint staining of the cytoplasm, loss of cell membrane outline, and loss of nuclear definition. Other histopathological examinations of the human temporal bone also demonstrated that mtDNA A3243G mutation can involve not only the stria vascularis and hair cells but also the spiral ganglion cells [22,23].

A defect in the inner hair cells, the auditory nerve, the connection between them, or the connection between the nerve and brain can lead to auditory neuropathy spectrum disorder (ANSD). ANSD has been reported to be associated with head injury; infections due to various viruses such as measles, mumps, and cytomegalovirus; and high fever and is also caused by specific gene mutations, such as OTOF. ANSD is characteristic of relatively mild hearing impairment with abnormal ABR response and poor speech recognition score, while distortion product otoacoustic emission (DPOAE) is normal [24,25]. In a report from Leruez et al. [26], 8 out of 19 patients with OPA1 gene mutation were reported to have suspected ANSD. Sakai et al. [27] reported a patient with normal DPOAE who had fluctuation of hearing threshold measured by ABR; because the peak latency of wave I and wave V and the intervals of waves I–V were markedly delayed, the existence of a retrocochlear problem was speculated to be a cause of hearing loss.

Cochlear implantation (CI) for patients with severe to profound hearing loss associated with mtDNA mutations has been reported [19,21,22,23,28,29,30,31,32,33,34,35]. Howes et al. [36] reported a case of MIDD with a speech score of 67% at one-month follow-up. Yasumura et al. [37] reported a case of MELAS with a speech score of 72% at 3-month follow-up. Li et al. [34] reported a case of MNGIE with a speech score of 56% at 3-month follow-up. All of these reports emphasized that CI is generally effective for patients with mtDNA mutations, but most of them only described speech perception in the short-term postoperative period. Therefore, it is unclear whether the effectiveness of CI is limited by the progression of retrocochlear dysfunction and/or cognitive decline associated with mitochondrial disorder. In fact, a patient has been reported to show poor postoperative speech perception associated with cognitive problems in relatively long-term follow-up [38].

In the present study, we investigated not only short-term but also long-term speech perception after CI in patients with profound hearing loss associated with mtDNA mutation.

## 2. Materials and Methods

A retrospective chart review was performed on patients who had undergone CI at the Department of Otolaryngology and Head and Neck Surgery at the University of Tokyo Hospital from 1991 to 2019. Nine patients were diagnosed with mtDNA mutations via genetic testing, and the additional information extracted included the causative mutations, clinical types, clinical course, perioperative complications, and postoperative speech perception. The Fukuda version of the monosyllabic speech perception test was used to evaluate speech perception before and after CI. Speech performance in noise was evaluated in four patients, including two patients examined twice, using a CI-2004 Japanese open-set sentence test. Tests were performed in quiet, SN20 and SN10. A DPOAE test and a promontory stimulation test were performed to differentiate between retrocochlear and cochlear hearing loss. Cases with obvious decline in attention, executive function, learning/memory, language, perceptual/motor functions, and social cognitive functions during the examination or as reported by family members were considered to have cognitive deterioration. The present study was approved by the Regional Ethical Standards Committee of the Faculty of Medicine at the University of Tokyo (application number 2487) and was conducted in accordance with the tenets of the Declaration of Helsinki. Written informed consent was obtained from the patients for publication of this study.

## 3. Results

### 3.1. Patient Characteristics

The characteristics of the nine patients with mtDNA mutations who underwent CI are shown in Table 1. The mean age at CI was 45.0 ± 11.5 years (range, 22–64 years), and the mean observation period was 5.5 ± 4.2 years (range, 1–13 years). A3243G mutation was identified in seven patients and RRM2B mutation and A8296G mutation were each identified in one patient. Of the seven patients with A3243G mutation, six patients were diagnosed with MIDD, and one was diagnosed with MELAS. A patient with the RRM2B mutation was diagnosed with CPEO, and a patient with the A8296G mutation only had hearing loss. Among the subjects, there were no suspicious findings of cognitive decline preoperatively. No patients showed any response in DPOAE tests, indicating that hearing loss involved the cochlea. All patients except one (patient 7) showed good response in promontory stimulation tests, which indicates that the retrocochlear auditory pathway was markedly involved in patient 7 but not in others.

### 3.2. Surgical Findings

No complications were observed during the surgery in any patient. A CI24M (Cochlear^®^, Lane Cove, Australia) electrode was used in patients 1, 2, and 3; a CI24RE (Cochlear^®^) electrode in patients 4, 5, 6, 7, and 9; and a CI422 (Cochlear^®^) electrode in patient 8. As there were no malformed cochlear cases in this series, we chose the latest electrode at surgery. All patients received CI only in the unilateral ear. Full insertion of CI electrodes was achieved in all patients. Electrically evoked compound action potentials were detected in all electrodes in all patients.

### 3.3. Postoperative Speech Perception

**Figure 1:**
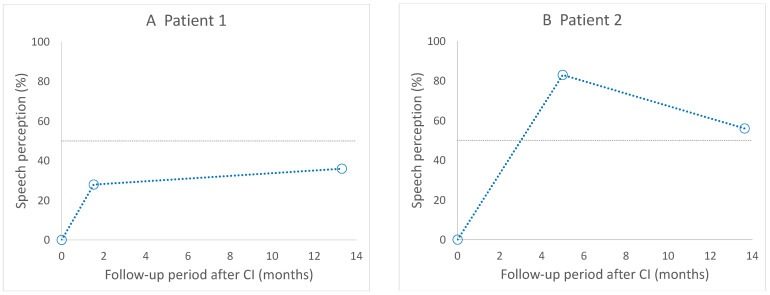

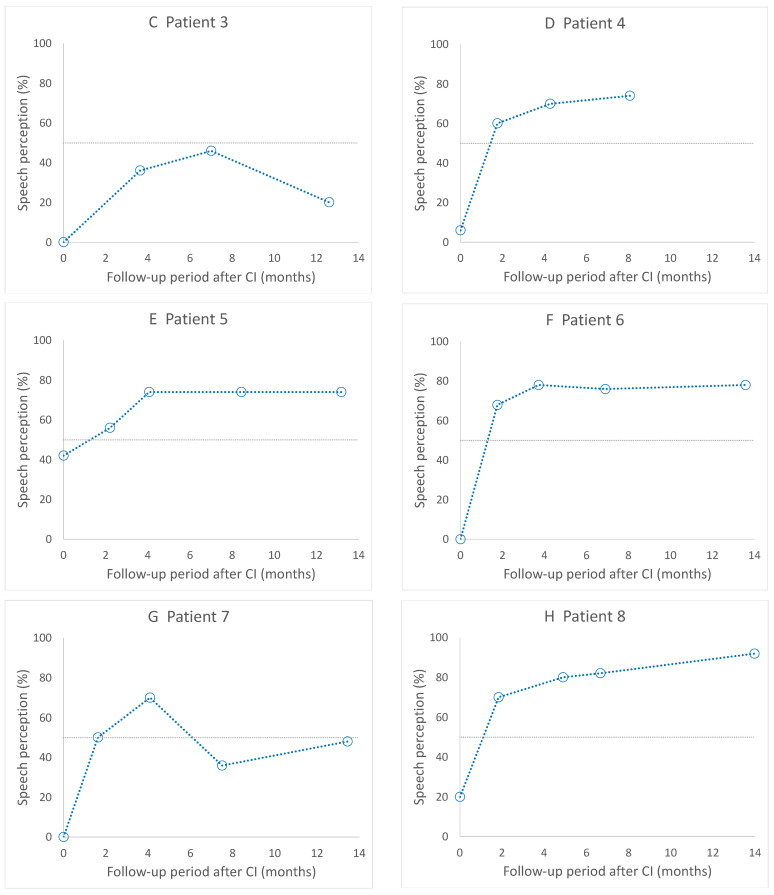

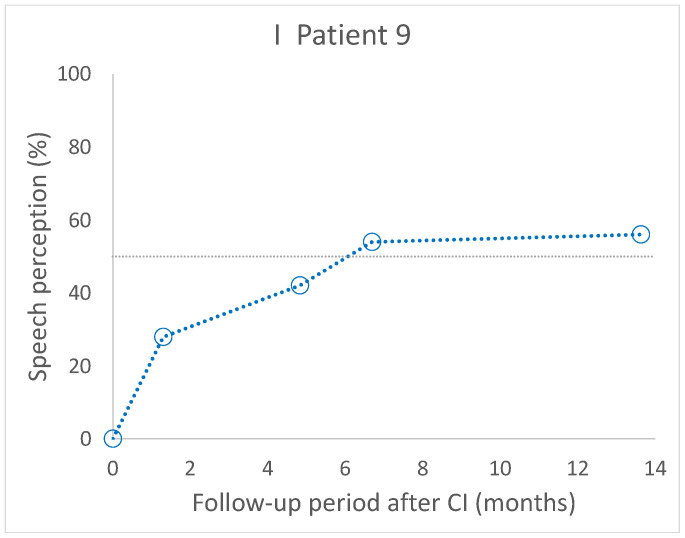
Short-term results of postoperative speech perception. Seven patients (**B**,**D**–**I**) achieved scores ≥ 50% after CI, and two patients (**A**,**C**) achieved poorer outcomes. CI, cochlear implantation.

Postoperative speech perception results within 14 months after CI are shown in Figure 1. Seven patients achieved scores of ≥50% in the Fukuda version of the monosyllabic speech perception test after CI, whereas two patients achieved scores of <50% (patients 1 and 3).

**Figure 2:**
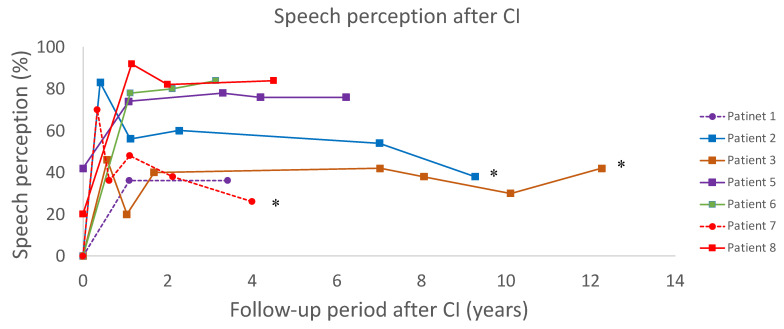
Long-term results of postoperative speech perception. Postoperative speech perception for seven patients. * Three patients with speech perception reduced by 20% or more.

The results of long-term postoperative speech perception are shown in Figure 2. Of the seven patients who were followed for more than 3 years, three patients (patients 2, 3, and 5) were followed for more than 5 years. Three patients (patients 2, 3, and 7) showed a decrease in postoperative speech perception of 20% or more. Patient 2 had no identifiable reasons for an acute deterioration in the first year and a gradual deterioration during the long-term follow-up. There was no sign of device failure, such as increasing impedances, an increase in clinical threshold level, or a reduced number of available electrodes in the course of deterioration of speech perception. In patient 3, a temporal shift in speech perception improved after mapping modification, and thereafter, no changes were observed during the long-term follow-up period. In patient 7, an acute deterioration in the first year was attributed to high-order brain dysfunction caused by cerebral infarction, but this episode did not cause the limited usage of the implant or make it difficult to conduct a speech perception test. After this episode, she showed a progressive decline in speech perception, despite the absence of an additional central episode or cognitive decline. There was no sign of device failure, such as increasing impedances, increases in threshold level by NRT, increases in clinical threshold level, or a reduced number of available electrodes in the course of deterioration of speech perception.

The results of sentence recognition tests in noise in four patients are shown in Table 2. Noise significantly influenced speech perception in one patient (patient 4), showing a poor score even in quiet conditions; this patient showed a progressive decline in the monosyllabic speech perception test. The other three patients maintained good scores under noise exposure, and two of them, who were examined twice using a sentence recognition test in noise, showed stable performance for more than three years; these patients also showed stable performance in long-term monosyllabic speech perception tests.

## 4. Discussion

In the present study, we investigated the short-term and long-term postoperative speech perception in nine patients who underwent CI for profound hearing loss with mtDNA mutation. Seven patients exhibited a good score of ≥50% in the Fukuda version of the monosyllabic speech perception test during the first postoperative year, but two of the seven patients showed deterioration during the long-term follow-up period. The short-term results in the current study agree with those in previous reports. Sinnathuray et al. [6] compared the results of postoperative speech perception in 12 patients with mtDNA mutations from 1997 to 2002 and reported good results irrespective of disease type, severity, and duration of hearing loss. Nawal et al. [39] conducted a systematic review of cochlear implantation outcomes in patients with mitochondrial hearing loss. In that study, 13 patients from 11 studies performed speech perception tests, and 10 out of 11 patients scored more than 50% in either speech, word, or phoneme recognition tests.

Deafness associated with mtDNA abnormalities is mainly attributed to dysfunction of the inner ear [11,12,13,14,15], but the retrocochlear auditory pathway may also be involved [4,16,17,21,22,23]. Short-term improvements in speech perception after CI may wane during long-term follow-up due to the degeneration of the spiral ganglion cells or cognitive decline due to progressive mitochondrial disorder. In a recently reported retrospective case series of five patients with mitochondrial diseases, including MELAS and MIDD, speech perception was preserved during the long-term follow-up period in four patients, but one patient could only use implants for several hours per day and could not conduct the speech perception test within 2 years of surgery [38]. In that report, the authors speculated that cognitive decline from the disease made the patient unable to recognize the importance of using the implant for the establishment of speech perception.

In the current study, two patients who initially achieved good speech perception exhibited a decrease in speech perception during the long-term follow-up period. In patient 2, neither cognitive decline nor deterioration of the device itself, such as a decrease in the number of available electrodes or an increase in the impedance of electrodes, were observed; therefore, progressive retrocochlear dysfunction was considered as the cause of the deterioration in speech perception. In another patient (patient 7), the initial decline in speech perception was associated with cerebral infarction, but the absence of additional central episodes, cognitive decline, or deterioration of the device itself thereafter suggests that the decline in speech perception during the long-term follow-up after cerebral infarction may be associated with progressive retrocochlear impairment associated with mtDNA mutation.

Previous studies [22,23,24,25,26,27,28,29,30,31,32,33,34,35,36,37,40,41] and short-term observations in the present study indicate that patients with mtDNA mutations are good candidates for CI. Notably, however, the long-term observations in the present study also suggest that retrocochlear dysfunction may be responsible for the long-term deterioration of speech perception after CI in patients with mtDNA mutations. Several reports [42,43,44] have investigated long-term speech performance in patients receiving CI and have observed no decline in speech perception performance. For example, Hilly et al. [42] examined 87 cochlear implant recipients, including 22 patients over 70 years of age, with a mean follow-up of 6.8 years, and found that most patients had a stable outcome during the follow-up period. Even in patients who are older, 13.6 percent improved and none had a reduction in score of more than 20 percent. Dillon et al. [43] followed 14 cochlear implant recipients aged 65 years and older for at least 10 years and found that consonant–nucleus–consonant word scores were stable between 6 months and 1 year of listening experience, improved significantly between 1 year and 5 years, and were stable between 5 years and 10 years. Hearing in Noise Test sentence scores in quiet and in noise showed a similar pattern, with stability in performance between the 6-month to 1-year and 5-year to 10-year follow-up intervals, and significantly improved performance between the 1-year and 5-year follow-up intervals. Therefore, diagnosis of mitochondrial diseases will have an impact on long-term performance of CI as well as future progression of hearing loss. Although CI has the potential to improve the quality of life in these patients, surgeons need to provide information about the possibility of gradual deterioration of speech perception in the long term after CI, so that patients and their families can prepare their future living environments and support. At our institution, we evaluate for mitochondrial genetic abnormalities at the time of initial consultation in patients with symptoms and signs suggestive of maternal inheritance.

Because no objective assessment data were available to confirm the progression of retrocochlear dysfunction, there were no clear predictors of the deterioration of speech perception during the long-term follow-up period. Preoperative diagnosis of retrocochlear involvement may have some impact on long-term performance of CI. All patients in our case series showed an absence of DPOAE response, indicating cochlear involvement. Absent or poor response in promontory stimulation tests indicates retrocochlear dysfunction. In the present study, two patients showed deterioration of speech performance during follow-up; one of them (patient 7) showed poor response in the promontory stimulation test, but the other (patient 2) showed good response. Therefore, it is unclear if preoperative retrocochlear involvement can predict the decline in speech perception in the long-term period. Breneman et al. [45] reported a long-term outcome of cochlear implantation in patients with auditory neuropathy spectrum disorder (ANSD). In that study, 35 patients with a follow-up period of more than six years on average showed as good a response as children with non-ANSD SNHL, which suggests that diagnosis of retrocochlear disease is not sufficient to predict the long-term benefit of CI. Superficial hemosiderosis is also known to present retrocochlear deafness. In a systematic review by Chaudhry et al. [46], 31 out of 44 patients showed improved hearing outcomes following CI, and 22 implants had sustained benefit at the last follow-up. They concluded that longevity of benefit was difficult to predict because of the progressive nature of the disease and a lack of preoperative prognosticators. Pijl et al. [41] used electrically evoked auditory brainstem response (EABR) and middle latency response (MLR) data derived from two patients with Kearns–Sayre syndrome and found that EABR and MLR were useful for distinguishing between cochlear and retrocochlear hearing loss and for predicting outcomes after CI. Rosenthal et al. [40] reported EABR and MLR data derived from a patient with MELAS syndrome who had significant central nervous system deficits, and proposed prioritizing MLR testing rather than EABR to evaluate the integrity of the auditory pathway. Introduction of these examinations may be useful for the prediction of future performance.

There have been several reports that older adult cochlear implant users have poorer performance of speech in noise compared to younger adults. This may be due to the fact that listening in noise is more susceptible to retrocochlear auditory pathway damage [47,48]. Although the present study could not provide sufficient data, it may be possible to predict the deterioration of speech performance with progression of retrocochlear dysfunction by repeated evaluation under noise conditions.

Generally, central nervous system symptoms, such as stroke-like episodes in MELAS patients, progress slowly [49,50]. Therefore, retrocochlear dysfunction after CI is also likely to progress slowly. Long-term observation may reveal deterioration of speech perception in our patients followed for less than 5 years.

It should be noted that heterogeneity of the presented samples may affect the interpretation of the results because of the relatively small number of cases. Although the time from deafness to surgery was around one year in most cases, there was diversity in the onset of hearing loss and the age of surgery. Heteroplasmy is also known to affect disease severity and the expression pattern of the impairment across organs and tissues [51,52], but was not analyzed in this study.

## 5. Conclusions

We retrospectively reviewed short-term and long-term speech perception after CI in nine patients with deafness associated with mtDNA mutations. Two of the seven patients who initially achieved good speech perception scores exhibited a deterioration in speech perception during the long-term follow-up. The absence of acute progression of cognitive decline in conjunction with the gradual decline in speech perception suggests that retrocochlear dysfunction associated with mitochondrial disorder could be responsible for the deterioration of speech perception.

## Figures and Tables

**Table 1:** Summary of patients.

Patient	Disease	Causative Mutations	Age of Onset of Hearing Loss (years)	Age of Becoming Deaf (years)	Age at CI (years)	Observation Period (years)	Associated Symptoms
1	MIDD	A3243G	30	53	53	3.4	diabetes
2	MIDD	A3243G	32	46	46	13	diabetes
3	MIDD	A3243G	38	44	44	12.2	diabetes
4	MIDD	A3243G	27	64	64	1.2	diabetes
5	MIDD	A3243G	10	50	51	6.2	diabetes
6	MIDD	A3243G	14	36	37	3.1	diabetes
7	MELAS	A3243G	10	44	46	4.0	myopathy, lactic acidosis, stroke-like episode
8	−	A8296G	7	21	22	4.5	-
9	CPEO	RRM2Bs	5	43	43	2.2	mild external ophthalmoplegia

Abbreviations: MIDD, maternally inherited diabetes with deafness; MELAS, mitochondrial myopathy, encephalopathy, lactic acidosis, and stroke-like episodes; CPEO, chronic progressive external ophthalmoplegia; CI, cochlear implantation.

**Table 2:** Results of speech in noise test.

Patient	Tested Year (Years after CI)	In Quiet (%)	S/N20 (%)	S/N10 (%)
4	5.2	45	20	-
5	1.3	95	95	58
	3.9	100	95	78
6	1.5	98	97	73
8	1.5	98	100	57
	3	100	92	-

A CI 2004 speech test was conducted in four patients at some point after CI. CI, cochlear implantation.

## Data Availability

The data presented in this study are available from the corresponding author upon request.
